# Supplementary material for: Rapid Removal of Toxic Remazol Brilliant Blue-R Dye from Aqueous Solutions Using Juglans nigra Shell Biomass Activated Carbon as Potential Adsorbent: Optimization, Isotherm, Kinetic, and Thermodynamic Investigation
Source: Int J Mol Sci. 2022 Oct 18;23(20):12484. doi: 10.3390/ijms232012484 (PMC9604326; doi:10.3390/ijms232012484)
Supplement: Supplementary file 1 [file ijms-23-12484-s001.zip › ijms-1871360-supplementary.pdf]

International Journal of
Molecular Sciences

MDPI

Article

Rapid Removal of Toxic Remazol Brilliant Blue-R Dye from Aqueous Solutions using *Juglans nigra* Shell Biomass Activated Carbon as Potential Adsorbent: Optimization, Isotherm, Kinetic, and Thermodynamic Investigation

Vairavel Parimelazhagan [1]*, Pranesh Yashwath [1], Dharun Arukkani Pushparajan [1] and Jitendra Carpenter [1]*

Supplementary Materials

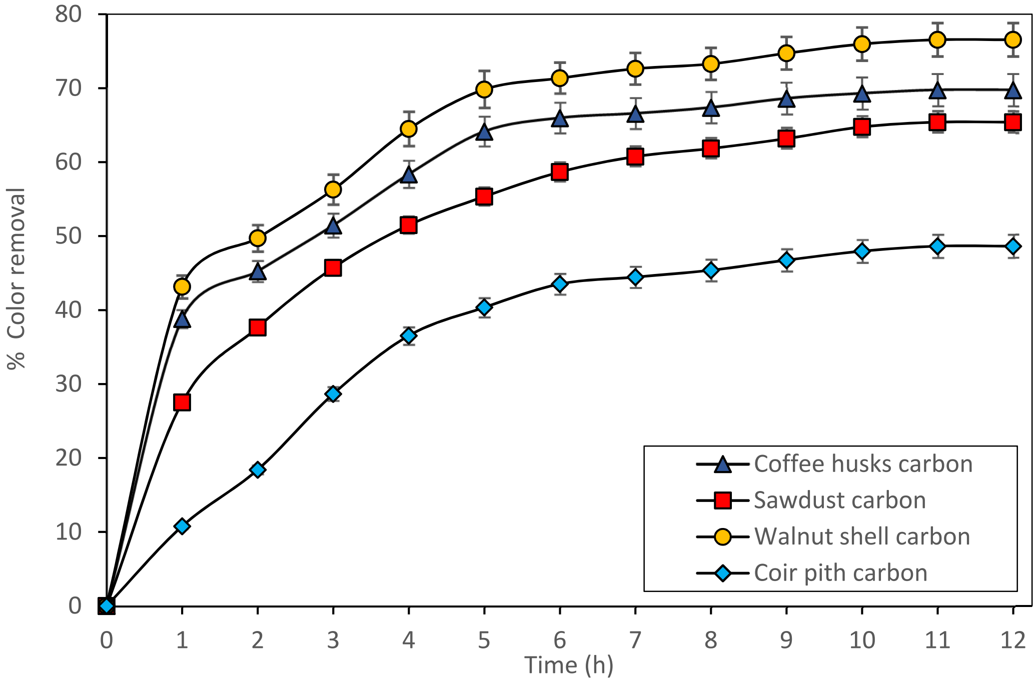

Figure S1. Selection of suitable agricultural biomass carbon for the decolorization of Remazol Brilliant Blue-R (RBBR) dye. (Initial pH: 2; initial dye concentration: 150 mg L^{-1}; agricultural biomass carbon adsorbent dosage: 4 g L^{-1}; adsorbent particle size: < 200 µm; agitation speed: 150 rpm; temperature: 301 K; contact time 12 h).

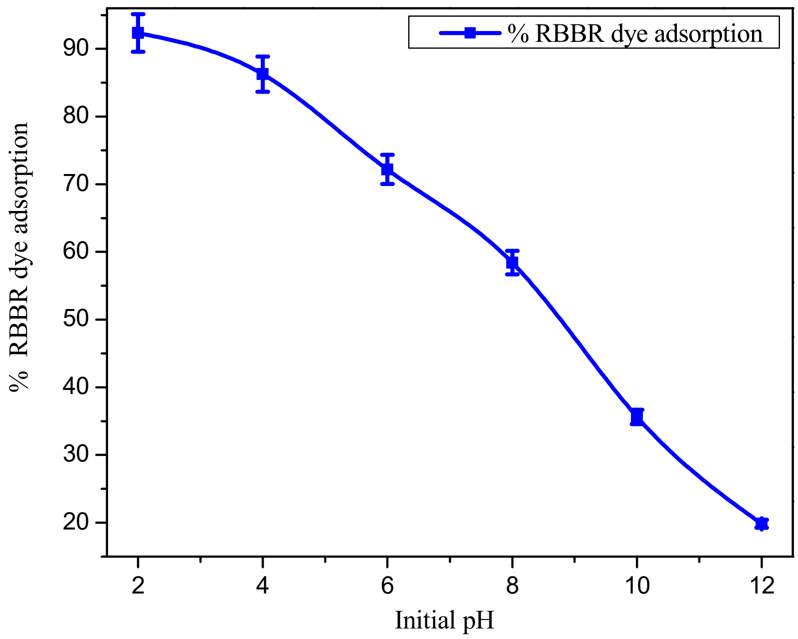

Figure S2. Effect of initial pH on removal of RBBR dye by walnut shell biomass activated carbon (WSBAC) adsorbent. (Initial dye concentration: 150 mg L^{-1}; WSBAC adsorbent dosage: 5 g L^{-1}; adsorbent particle size: 124 µm; agitation speed: 150 rpm; temperature: 301 K; contact time: 8 h).

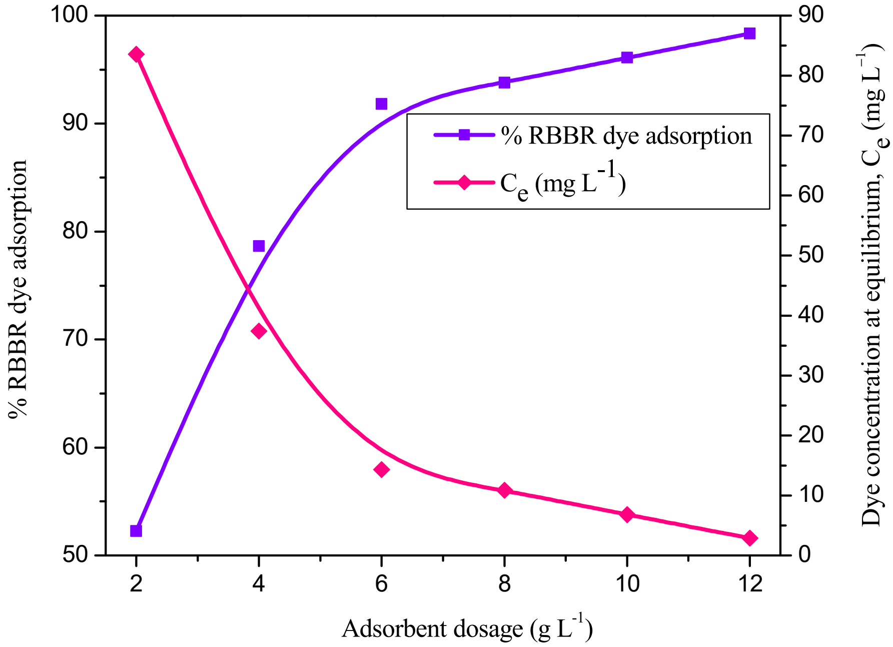

Figure S3. Effect of WSBAC adsorbent dosage on RBBR dye decolorization. (Initial pH: 2; initial dye concentration: 175 mg L^{-1}; WSBAC adsorbent particle size: 124 µm; agitation speed: 150 rpm; temperature: 301 K; contact time: 24 h).

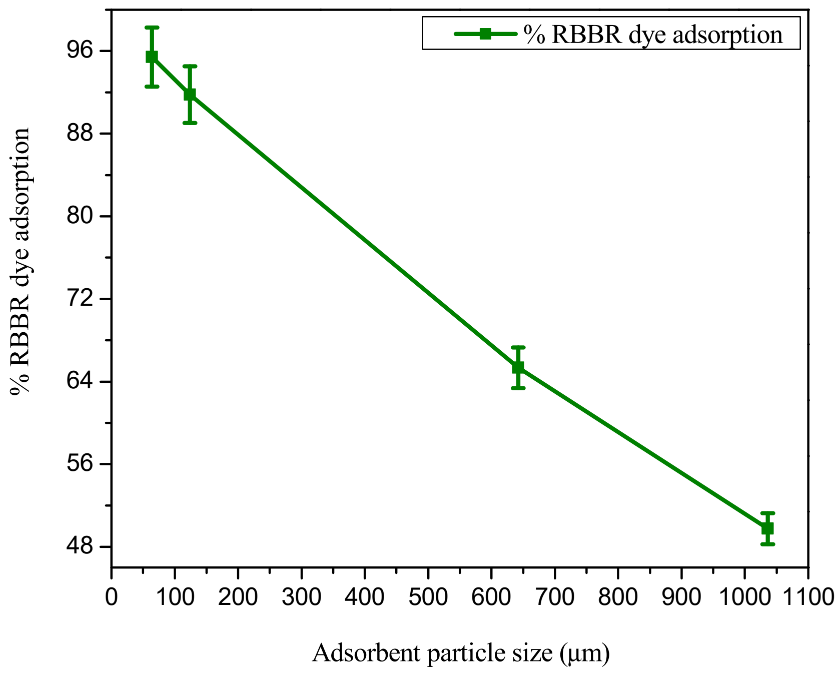

Figure S4. Effect of WSBAC adsorbent particle size on RBBR dye adsorption. (Initial pH: 2; initial dye concentration: 175 mg L^{-1}; WSBAC adsorbent dosage: 6 g L^{-1}; agitation speed: 150 rpm; temperature: 301 K; contact time: 8 h).

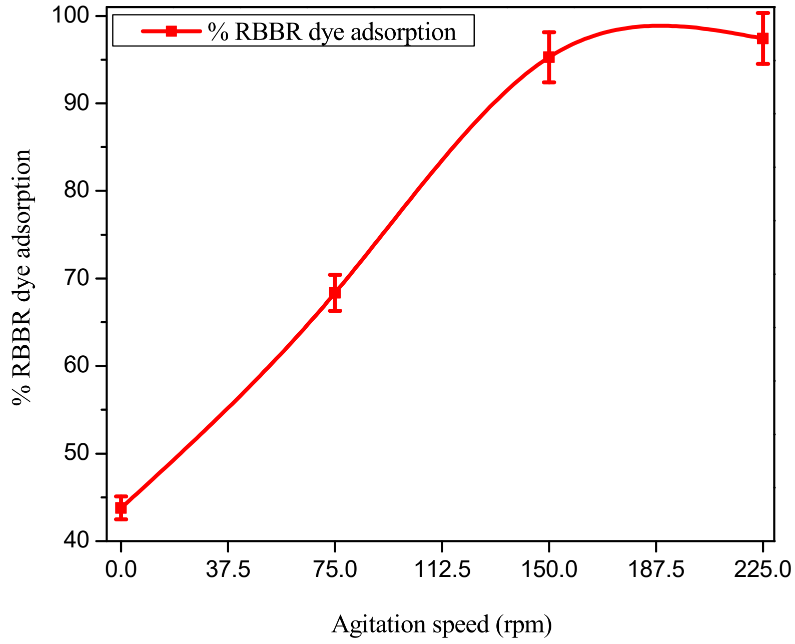

Figure S5. Effect of agitation speed on the removal of RBBR dye by WSBAC adsorbent. (Initial pH: 2; initial dye concentration: 175 mg L^{-1}; WSBAC adsorbent dosage: 6 g L^{-1}; adsorbent particle size: 65 µm; temperature: 301 K; contact time: 8 h).

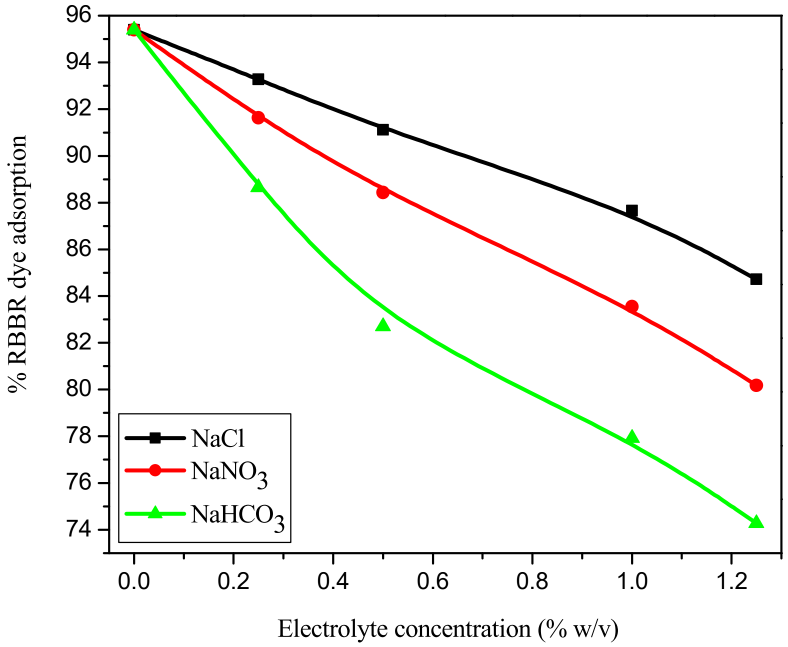

Figure S6. Effect of electrolytes on decolorization of RBBR dye using WSBAC adsorbent. (Initial pH: 2; initial dye concentration: 175 mg L^{-1}; WSBAC adsorbent dosage: 6 g L^{-1}; adsorbent particle size: 65 µm; agitation speed: 150 rpm; temperature: 301 K; contact time: 8 h).

(**a**) (**b**)

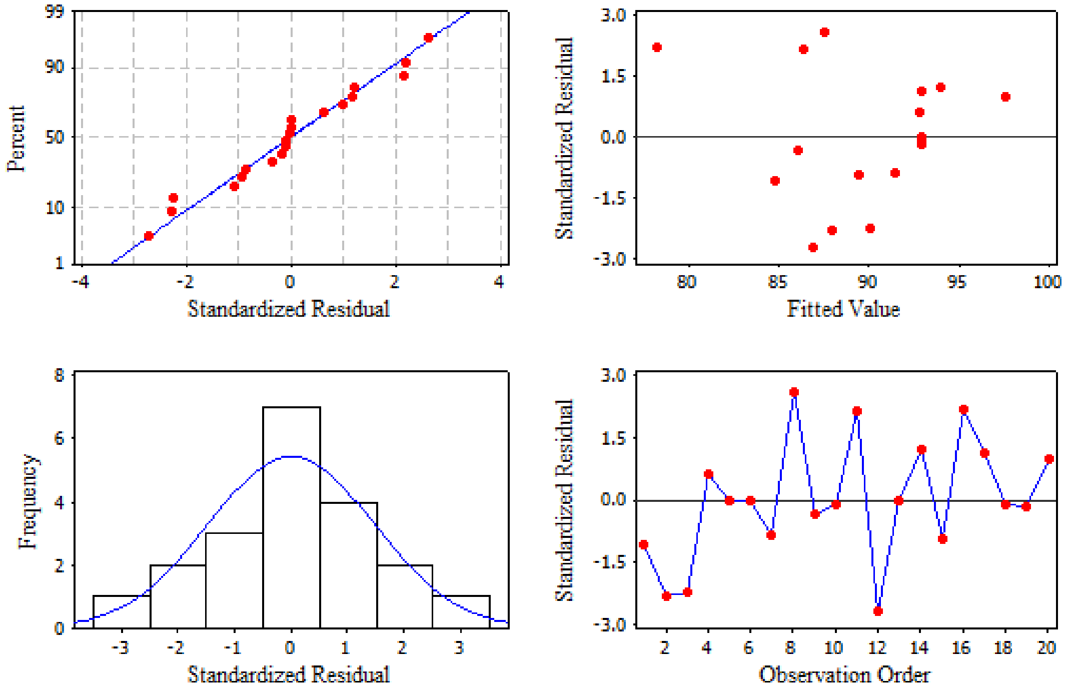

(**c**) (**d**)

Figure S7. Residual plots for RBBR color removal by WSBAC adsorbent. (a) Normal probability plot of standardized residuals, (b) Standardized residuals vs. fitted values, (c) Frequency of observation vs. standardized residuals, and (d) Standardized residuals vs. order of the data.

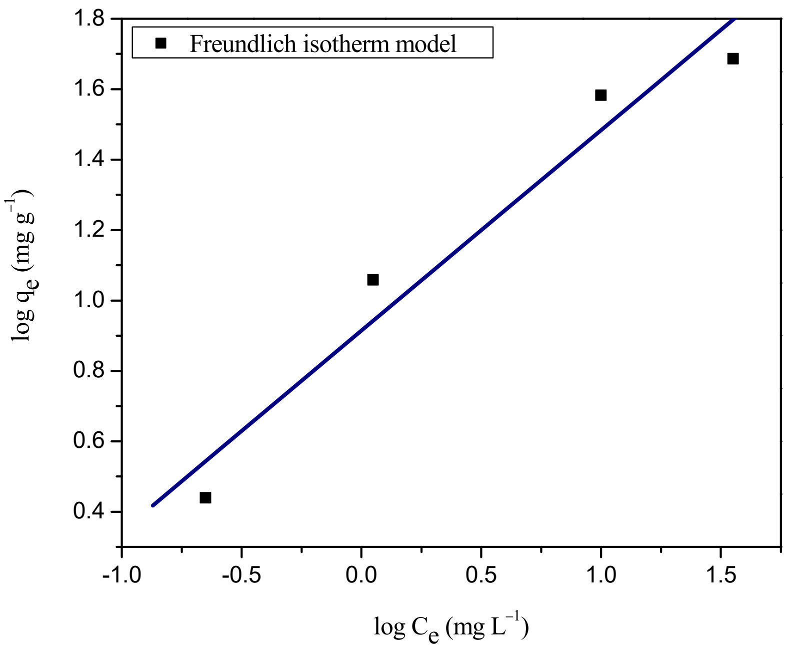

Figure S8. Freundlich isotherm plot for RBBR dye adsorption onto WSBAC adsorbent. (Initial pH: 1.5; initial dye concentration: 25–225 mg L^{-1}; WSBAC adsorbent dosage: 6 g L^{-1}; adsorbent particle size: 55.21 μm; agitation speed: 150 rpm; temperature: 301 K; contact time: 24 h)

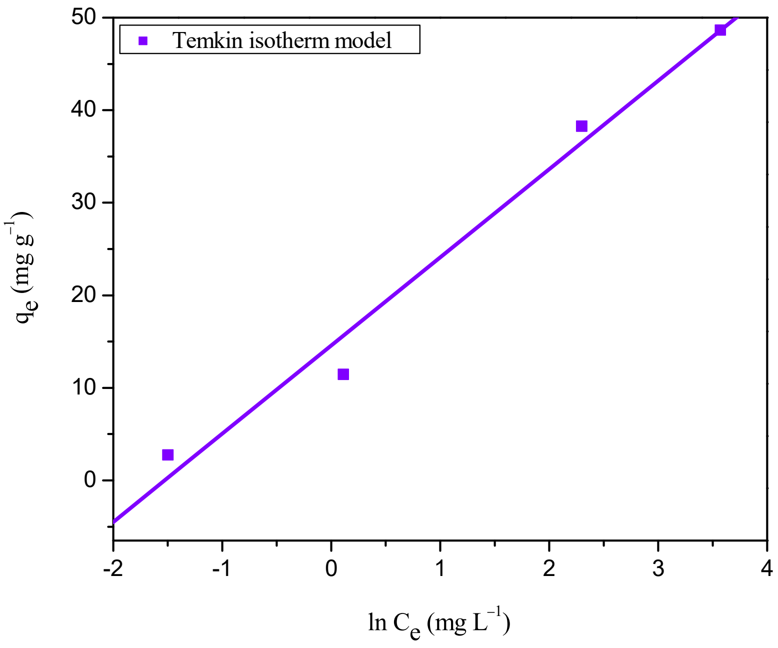

Figure S9. Temkin isotherm plot for RBBR dye adsorption onto WSBAC adsorbent. (Initial pH: 1.5; initial dye concentration: 25–225 mg L^{-1}; WSBAC adsorbent dosage: 6 g L^{-1}; adsorbent particle size: 55.21 μm; agitation speed: 150 rpm; temperature: 301 K; contact time: 24 h).

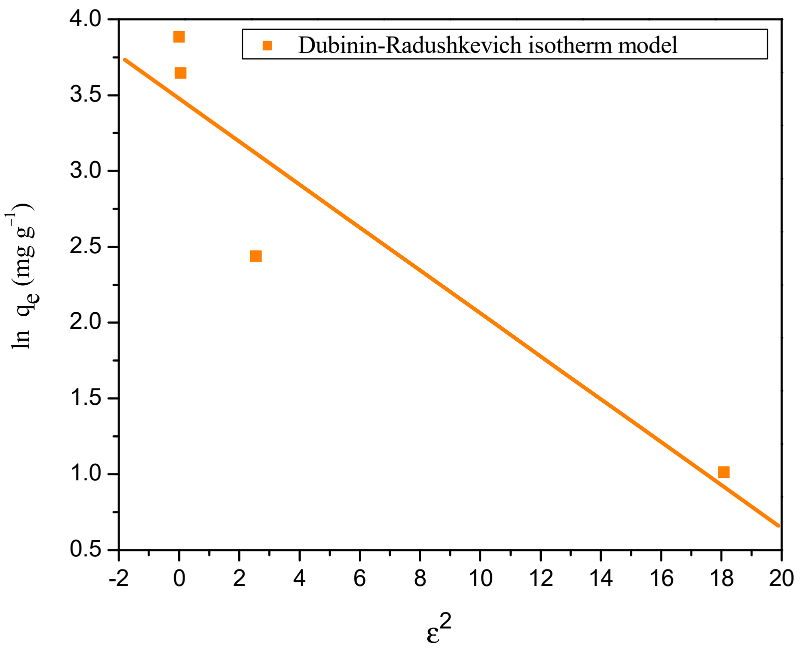

Figure S10. Dubinin-Radushkevich isotherm plot for RBBR dye adsorption onto WSBAC adsorbent. (Initial pH: 1.5; initial dye concentration: 25–225 mg L^{-1}; WSBAC adsorbent dosage: 6 g L^{-1}; adsorbent particle size: 55.21 μm; agitation speed: 150 rpm; temperature: 301 K; contact time: 24 h).

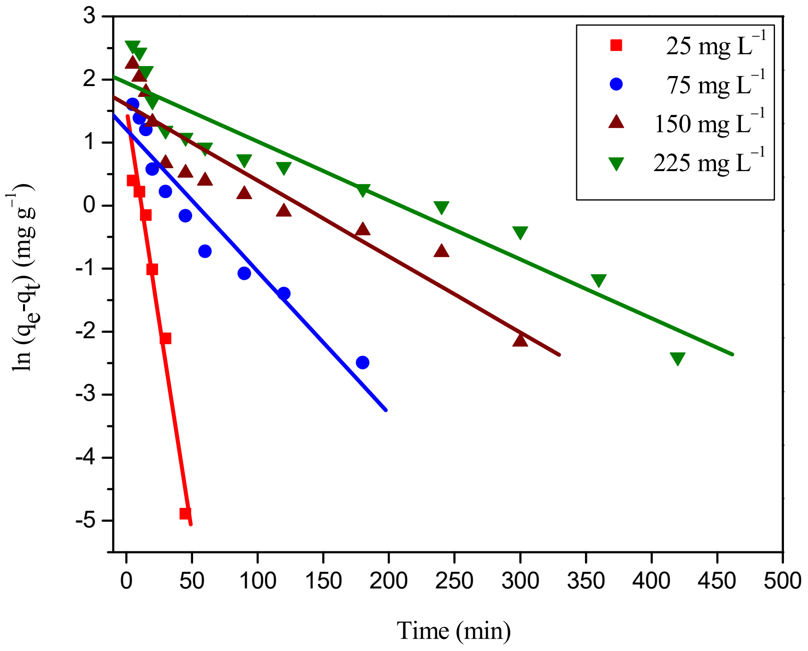

Figure S11. Lagergren pseudo-first-order kinetic plot for adsorption of RBBR dye onto WSBAC adsorbent. (Initial pH: 1.5; initial dye concentration: 25–225 mg L^{-1}; WSBAC adsorbent dosage: 6 g L^{-1}; adsorbent particle size: 55.21 μm; agitation speed: 150 rpm; temperature: 301 K; contact time: 24 h).

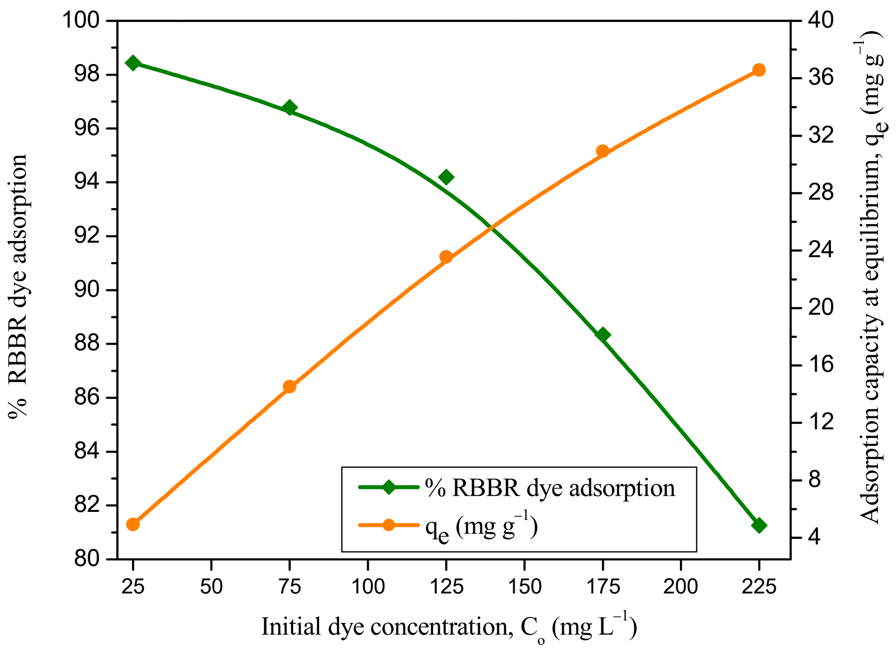

Figure S12. Effect of initial dye concentration on the removal of RBBR dye by WSBAC adsorbent. (Initial pH: 2; WSBAC adsorbent dosage: 5 g L^{-1}; adsorbent particle size: 124 μm; agitation speed: 150 rpm; temperature: 301 K; contact time: 24 h)

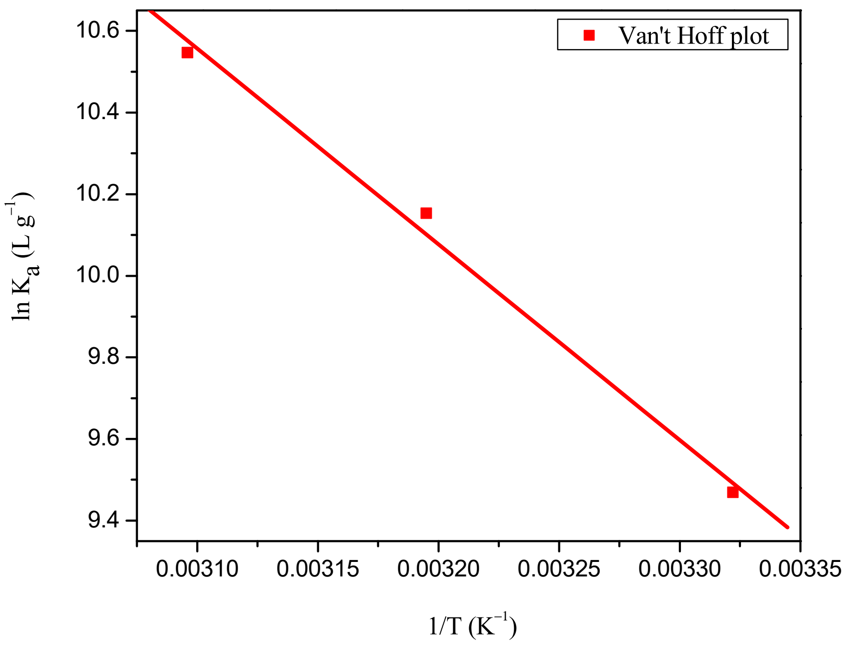

Figure S13. Van't Hoff plot for decolorization of RBBR dye using WSBAC adsorbent. (Initial pH: 1.5; initial dye concentration: 25–225 mg L^{-1}; WSBAC adsorbent dosage: 6 g L^{-1}; adsorbent particle size: 55.21 μm; agitation speed: 150 rpm; contact time: 24 h).

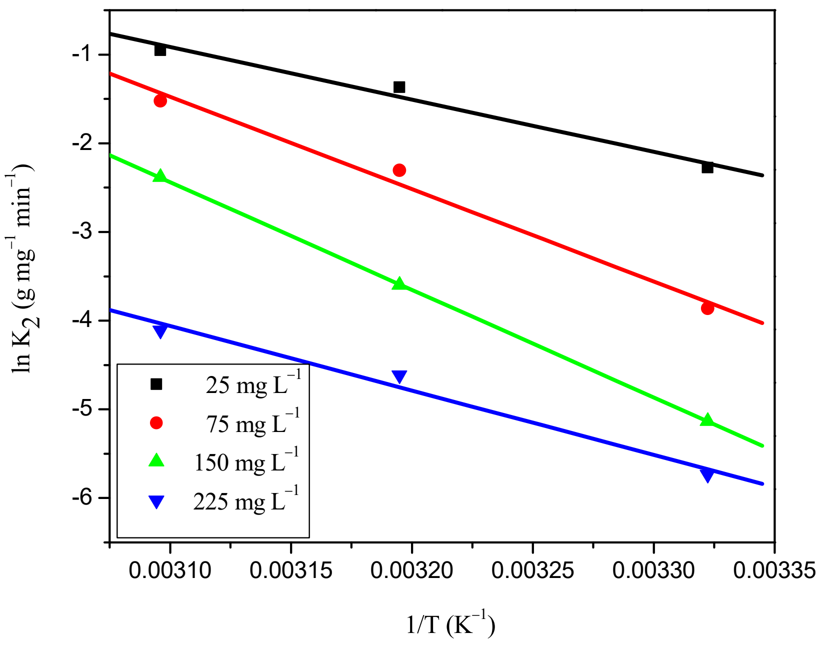

Figure S14. Arrhenius plot for decolorization of RBBR dye using WSBAC adsorbent. (Initial pH: 2; initial dye concentration: 25–225 mg L^{-1}; WSBAC adsorbent dosage: 6 g L^{-1}; adsorbent particle size: 55.21 µm; agitation speed: 150 rpm; contact time: 24 h).

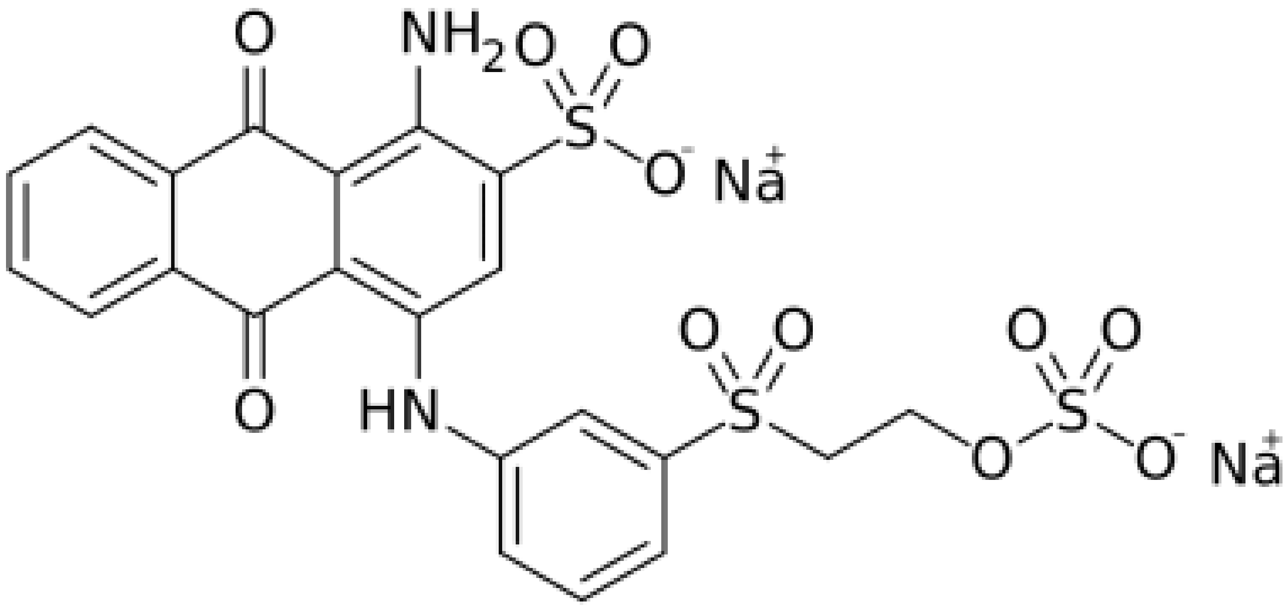

Figure S15. Chemical Structure of Remazol Brilliant Blue –R (RBBR) dye.

Table S1. Energy Dispersive X-ray Spectroscopy(EDS) analysis of WSBAC adsorbent before and after RBBR dye adsorption.

Process mode	Elements (weight %)					Elements (atom %)				
	C	O	Na	N	S	C	O	Na	N	S
Before adsorption	1.92	4.83	0	0	0	43.84	56.16	0	0	0
After adsorption	2.89	6.25	0.08	0.54	0.24	56.34	82.44	1.05	5.12	1.65

Table S2. Elemental analysis of raw walnut shell biomass.

Elements	Weight %	Atom%
C	49.83	42.16
O	45.24	51.64
N	2.17	1.84
P	1.08	1.65

Table S3. Activation energy for the adsorption of RBBR dye onto WSBAC adsorbent at various initial dye concentrations.

Initial dye concentration, C_o (mg L^{-1})	Activation energy, E_a (kJ $mole^{-1}$)
25	49.092
75	60.384
150	86.589
225	100.935
